# Glucose Sensing by Time-Resolved Fluorescence of Sol-Gel Immobilized Glucose Oxidase

**DOI:** 10.3390/s110403483

**Published:** 2011-03-24

**Authors:** Rosario Esposito, Bartolomeo Della Ventura, Sergio De Nicola, Carlo Altucci, Raffaele Velotta, Damiano Gustavo Mita, Maria Lepore

**Affiliations:** 1 CNISM-Dipartimento Scienze Fisiche, Università di Napoli Federico II, Napoli 80126, Italy; E-Mails: altucci@na.infn.it (C.A.); velotta@na.infn.it (R.V.); 2 Dipartimento di Medicina Sperimentale, Seconda Università di Napoli, Napoli 80138, Italy; E-Mails: dellaventura@na.infn.it (B.D.V.); mita@igb.cnr.it (D.G.M.); maria.lepore@unina2.it (M.L.); 3 Istituto Nazionale di Ottica, CNR, compr. A. Olivetti, Via Campi Flegrei 34, Pozzuoli 80078, Italy; E-Mail: s.denicola@cib.na.cnr.it; 4 Institute of Genetics and Biophysics, CNR, Naples 80131, Italy

**Keywords:** glucose oxidase, biosensors, glucose determination, time-resolved fluorescence, lifetime, **PACS** 87.85.fk, 87.64.kv, 78.47.D-, 33.70.Ca

## Abstract

A monolithic silica gel matrix with entrapped glucose oxidase (*GOD*) was constructed as a bioactive element in an optical biosensor for glucose determination. Intrinsic fluorescence of free and immobilised *GOD* was investigated in the visible range in presence of different glucose concentrations by time-resolved spectroscopy with time-correlated single-photon counting detector. A three-exponential model was used for analysing the fluorescence transients. Fractional intensities and mean lifetime were shown to be sensitive to the enzymatic reaction and were used for obtaining calibration curve for glucose concentration determination. The sensing system proposed achieved high resolution (up to 0.17 *mM*) glucose determination with a detection range from 0.4 *mM* to 5 *mM*.

## 1. Introduction

In the last years the employment of glucose oxidase (*GOD*) in glucose optical sensing has been largely investigated for clinical and industrial applications [1–8]. Different immobilization procedures have been adopted [9–11] aiming to improve the linear range, sensibility, specificity, reproducibility and time stability of the optical sensors. Sol-gel matrices have been proven to be excellent carriers for enzymes since their catalytic activity is slightly affected by the gel structure. In addition sol-gel avoids the leakage of the bioactive macromolecule and allows the diffusion of substrate (and reaction products) towards (or away from) the catalytic site [12–14].

**Figure 1.**
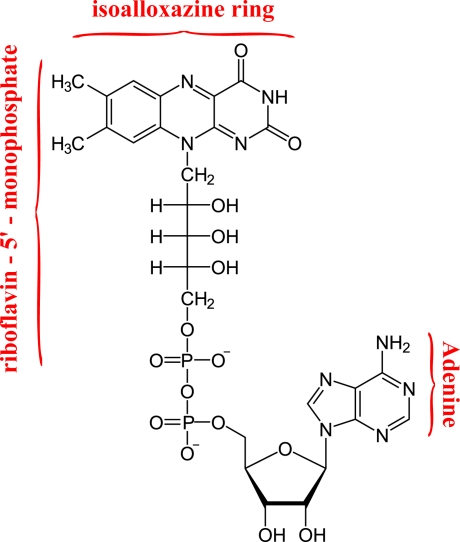
Structural formula of flavin adenine dinucleotide (*FAD*).

It is well known that *GOD* is a homo-dimer composed of two identical 80-kDa subunits and two non-covalently bound flavin adenine dinucleotides complexes (*FAD*). *FAD* is situated in the first domain which consists of two beta sheets and three alfa helices and occupies a narrow channel outlined by 31 residues with a covering lid formed by residues 75–98 [15]. The *FAD* structure is composed by flavoprotein and adenine dinucleotide (Figure 1). The isoalloxazine (*ISO*) ring is responsible for the light emission of *FAD* in the visible spectral range and is linked with adenine through hydrogen-bonding. It was proposed that *FAD* exists in two conformations: an extended, open conformation, and a closed one, in which the isoalloxazine and adenine rings interact through a stacked conformation. The open conformation gives rise to the fluorescent component of *FAD* with lifetime ∼2.8 ns, whereas a much faster fluorescence decay (∼4 ps) takes place as a consequence of the photoinduced electron transfer (ET) between adenine and flavin [16,17]. The conformational changes experienced by the molecule during the lifetime of the excited states accounts for an intermediate lifetime component of ∼0.3 ns.

In flavins, photoinduced ET from adenine to excited *ISO* competes with the reductive ET mechanism that involves tyrosine and tryptophan residues adjacent to *ISO*. Particularly, X-ray crystallographic structure of *GOD* showed that four aromatic residues, Tyr515, Trp426, Tyr68, and Trp111, are in close contact with the isoalloxazine moiety of *FAD* and these residues serve as electron donors in the photoinduced reductive ET [18,19]. With femtosecond resolution, the fluorescence quenching of *GOD* due to ET with aromatic residues has been investigated and two decay constants of 1.8 ps and 10 ps have been measured [20]. Thus, we can assume three ranges for fluorescence lifetimes in flavins: long (∼2.0–3.0 ns), intermediate (∼300 ps) and short (≤10 ps).

Dynamical changes of *FAD* fluorescence are exhibited when oxidative processes are induced in *GOD* by means of glucose interaction [21–23]. In fact, *GOD* catalyses the oxidation of glucose to gluconic acid through the reaction:

$$\begin{array}{l} D-\mathrm{glucose}+0_{2}\overset{\mathrm{GOD}}{\to }D-\text{gluconolactone}+H_{2}0_{2}\\ D-\text{gluconolactone}+H_{2}0\to D-\mathrm{gluconic acid} \end{array}$$

**Figure 2.**
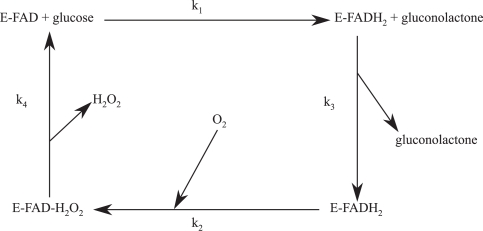
Scheme of the enzymatic reaction. *O*_2_ is the oxygen concentration in the bulk solution.

The well-known reaction mechanism is the following: glucose reduces *FAD* to *FADH*_2_ with formation of gluconolactone, which is rapidly hydrolysed to gluconic acid. At this point the dissolved oxygen reoxidises *FADH*_2_ and produces *H*_2_*O*_2_. According to the cyclic scheme of Figure 2, two species display the fluorescence of *FAD* and *FADH*_2_.

The chemical reduction of *FAD* to *FADH*_2_ should affect the reductive photoinduced ET interaction with the surrounding aromatic residues. The glucose and oxygen concentrations and the induced change of the pH environment could contribute to alter the usual equilibrium between the normal and closed conformations of *FAD*, thus causing a change in the fluorescence intensity [24]. In Reference [25] some of us have investigated on the possibility of using that change in *GOD* immobilised in a silica gel matrix for glucose based biosensor. To this regard, physicochemical and biochemical characterizations of the catalytic matrix have been performed and the intrinsic fluorescence of immobilised *GOD* has been investigated by steady-state fluorescence measurements. The silica gel matrix has been proved to be a suitable support for optical biosensing owing to its superior optical properties such as high transmittance, reproducibility of *GOD* fluorescence and absorption spectra after immobilization. Moreover, calibration curves as a function of glucose concentration have been obtained by steady-state measurements with sensitivity and linear calibration range comparable to the other immobilization systems.

However, it has been shown [26] that the use of decay times, as opposed to intensities, is preferred in view of their independence on probe concentrations as well as the intensity fluctuations of the fluorescence signal. Moreover, lifetimes can be measured also in highly scattering media [27,28]. Infact, successful measurements through several layers of chicken skin have been performed, giving a promising issue for glucose sensing in real samples like tissue and blood [29].

In this paper we investigate the dynamic behaviour of interactions between free *GOD* and glucose and the modifications occurring when *GOD* is immobilized in the silica gel matrix used in Reference [25]. We use time-resolved fluorescence for accurate measurements of fractional intensities and lifetimes as sensitive parameters to the enzymatic reaction of *GOD* with glucose and we show that it is possible to achieve improvements of the performances of the sensing system.

The outline of the paper is the following. In Section 2 we describe the materials and methods adopted to realize sol-gel matrices that immobilize *GOD*. Experimental setup used for steady-state and time-resolved measurements are also reported together with the multi-exponential model adopted to analyse decay curves. Steady state and time-resolved fluorescence measurements for free *GOD* without and with glucose are discussed in Section 3.1, and experimental results for immobilised *GOD* are analysed in Section 3.2. Moreover, steady-state and temporal profiles for free and immobilised cases are compared. In Section 3.3 the fractional intensities of lifetime components are retrieved by means of a fitting procedure and their behaviour are analysed for obtaining calibration curves as a function of glucose concentration. To conclude, the analytical performance of the proposed sensing system is compared with that of various *GOD* based optical biosensors in terms of the immobilization support, detection method, stability, linear range and detection limits.

## 2. Materials and Methods

### 2.1. Materials

All chemicals were purchased from Sigma (Sigma Italia, Milan, Italy), with the exception of tetramethoxysilane (TMOS) and glucose oxidase (*GOD*), which were purchased from Fluka (Fluka Italia, Milan, Italy). In this study glucose oxidase (*GOD*, EC 1.1.3.4) from Aspergillus niger (154 *U mg*^1^) was used.

### 2.2. Methods

#### 2.2.1. Preparation of the Catalytic Sol-Gel Matrices

Silica gel matrices were prepared, as described in a previous paper [9], by rapid mixing 800 *μL* of solution A with 800 *μL* of solution B in a polymethylmethacrylate cell. Solution A was obtained by slowly mixing (for 1 *h* at 4 °C) TMOS (1,550 *μL*) with *H*_2_*O* (450 *μL*) plus 40 *mM* HCl (30 *μL*). Solution B contained 0.1 M phosphate buffer at pH 6.5. The A and B solution mixture (1,600 *μL*) was poured into a polymethylmethacrylate cell that was sealed with paraffin film and placed horizontally until the gel was formed. To avoid cracking in the formed gel, the cell was filled with 0.1 *M* phosphate buffer at pH 6.5 and stored overnight in a refrigerator at 4 °C. The day after, the silica gel layer was removed from the cell and was ready for use. The catalytic gel was obtained with solution B containing an additional 20 *mg/mL* of *GOD* in 0.1 *M* phosphate buffer at pH 6.5.

Generally, the retention of *GOD* activity after entrapment in silica gel is very low since the entrapment of *GOD* changes the properties of the enzyme, especially its stability [30]. The change in the properties of the enzyme inside the gel is due to the change in its conformation leading sometimes to a denaturation of protein, restriction of molecule motion and lower accessibility of the entrapped enzyme by the substrate [14]. The changes of protein conformation and dynamic motion inside the gel are caused by interactions of the molecule with silica surface inside the pores and different micro-environment. As a result, the activity of the entrapped enzyme is typically lower and the stability can either be decreased or increased depending on the composition of the gel [14]. The properties of *GOD* immobilised in the proposed silica gel have been extensively investigated in Reference [25]. It results an apparent value of Michaelis–Menten constant larger than that obtained for free *GOD* as a result of its lower activity. Moreover, the investigations have evidenced a high catalytic stability of the gel since only a decrease of about 2% for the absorbance spectrum has been observed after 60 days. In the following all the measurements have been performed at room temperature.

#### 2.2.2. Steady-State Fluorescence Measurements

The emission fluorescence spectra have been collected by means of a spectrofluorimeter (model LS 55, Perkin-Elmer), by using as light source a Xenon discharge lamp with an emission spectrum ranging from 200 to 800 *nm*. Sample excitation was performed at 450 *nm*, while the emission spectrum was recorded in the range 480–580 nm. The spectra have been acquired with entrance and exit slit fixed at 5 *nm* and with a scan speed of 100 *nm s^−^*^1^.

#### 2.2.3. Time-Resolved Fluorescence Measurements

**Figure 3.**
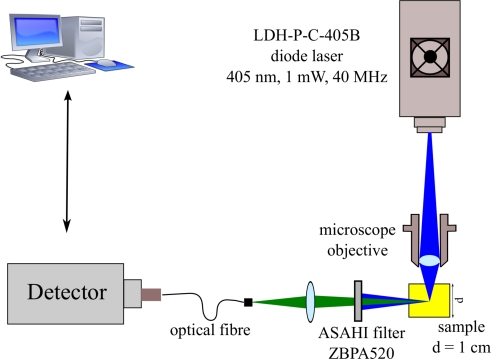
Experimental setup.

Time-resolved fluorescence experiments were carried out using the experimental setup shown in Figure 3.

Excitation for the solution was provided by a picosecond diode laser (Picoquant, LDH-P-C-405B) emitting pulses at repetition rate of 40 *MHz* and a wavelength of 405 *nm*, close to the absorption maximum of the first electronic transition of the bound flavin. The laser beam was focused into a 10 *mm* sample cell by a simple microscope objective lens. The fluorescence light was detected at 90° to the incident light beam to minimize the amount of transmitted or reflected beam light reaching the detector. An Asahi ZBPA520 bandpass filter blocks the residual laser beam and allows only radiation with a wavelength of 520 ± 10 *nm* to reach the detector, that is a wavelength range close to the maximum of fluorescence emission spectrum of *GOD*. The detection apparatus was composed of a fast multichannel plate photomultiplier tube (MCP-PMT) (R4110U-05-F008aA, Hamamatsu, Tokyo, Japan) and a time-correlated single-photon counting (TCSPC) electronics (SPC300, Becker and Hickl GmbH, Berlin, Germany). The instrument response function (IRF) determined by TCSPS was about 120 *ps* FWHM.

Analysis of fluorescence transients was performed through a fitting procedure with a three-exponential model, in agreement with the description of redox reaction proposed in References [16,20]. Effects of the system response have been taken into account by convolving the analytical model with IRF, according to the following expression:

(1) $$I(t)=\mathrm{IRF}(t)⊗\sum_{i=1}^{3}\frac{\alpha_{i}}{\tau_{i}}\exp(-\frac{t}{\tau_{i}})$$

where the pre-exponential factor *α_i_* is the total fluorescence intensity for the *i^th^* emitting component with lifetime *τ_i_*. The mean lifetime *τ̄* is obtained by the classical relationship:

(2) $$\bar{\tau }=\frac{\sum \alpha_{i}\tau_{i}}{\sum \alpha_{i}}=\sum f_{i}\tau_{i},$$

where *f_i_* are the fractional steady-state intensities of each lifetime component:

(3) $$f_{i}=\frac{\alpha_{i}}{\sum \alpha_{i}}$$

## 3. Results and Discussion

### 3.1. Free GOD

**Figure 4.**
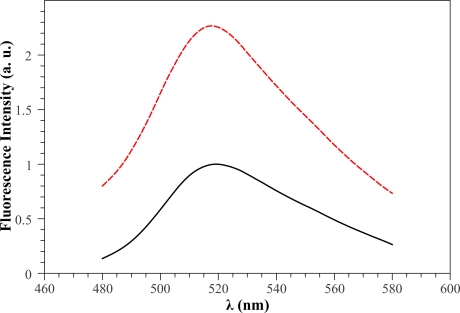
Free *GOD* fluorescence emission spectrum in the *FAD* region (λ*_exc_* = 450 *nm*) without (black solid curve) and with (red dotted curve) 1 *mM* glucose concentration.

As a preliminary step we examined the steady-state fluorescence properties of free *GOD* and the changes induced by glucose addition. According to the results reported in Reference [31], the maximum of the fluorescence emission of free *GOD* was at 520 *nm* when the excitation is at 450 *nm*. Generally, the effect of glucose addition is to increase the peak value and the area of the fluorescence intensity emission spectrum, since reduced and oxidized flavins (*FADH*_2_ and *FAD*) exhibit different fluorescent properties [24]. Figure 4 shows, as an example, the fluorescence emission spectra of soluble *GOD* in absence (black solid curve) and in the presence of 1 *mM* glucose concentration (red dotted curve). It can be clearly seen that the inclusion of glucose results in a fluorescence peak value that almost doubles with a corresponding increase of the integral area under the spectrum.

**Figure 5.**
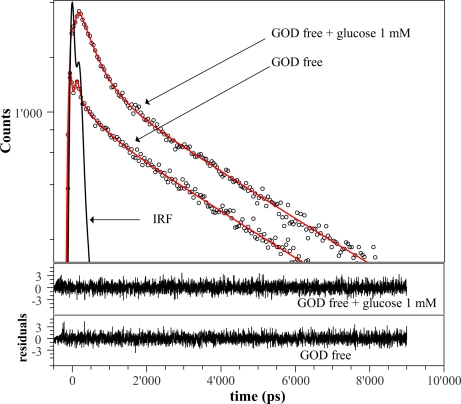
Black points represents the fluorescence decays for free *GOD* when excited in the FAD region (λ*_exc_* = 405 *nm*) without and with glucose (1 mM). Red solid curves are three-exponential fits. The instrument response function is shown for comparison with the fluorescence decay. In the lower panels residuals for three-exponential models are reported. The fitting parameters are shown in Table 1.

A better understanding of the fluorescence intensity enhancement is given by time-resolved measurements. To this regard, we have plotted in Figure 5 the decays curves (black points) for *GOD* in absence and in presence of 1 *mM* glucose concentration. The random distribution of residuals around zero confirms that the observed data can be satisfactorily fitted with the three-exponential model (1) (red solid curves). The estimated parameters are shown in Table 1 and their values are in quite good agreement with results reported in References [16,20] for free *GOD*.

The sum of fluorescence intensities, $\sum_{i=1}^{3}\alpha_{i}$, is comparable with intensities of steady-state measurements and increases to about 87% upon glucose addition. In particular, the values of fractional intensities *f_i_* reported in Table 1 are clear evidence of a fluorescence quenching of the fast and long component *τ*_1_ and *τ*_3_. The intensities *f*_1_ and *f*_3_ change from 0.054 to 0.015 and from 0.88 to 0.76, respectively. Conversely, the intermediate fluorescence component *f*_2_ increases from 0.06 to 0.22 accounting for the overall enhancement of the fluorescence intensity as glucose is added into the aqueous solution.

### 3.2. Immobilized GOD

**Figure 6.**
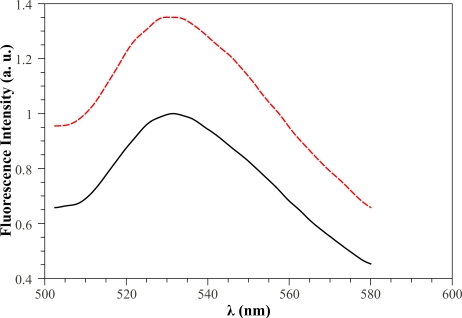
Immobilized *GOD* fluorescence emission spectrum in the FAD region (λ*_exc_* = 405 nm) without (black curve) and with (red curve) 2 *mM* glucose concentration.

The black curve of Figure 6 represents the fluorescence spectrum of *GOD* immobilized in a sol gel layer. Also in this case, the presence of glucose in solution has the effect of enhancing the fluorescence intensity as it is evidenced by the red curve shown in Figure 6 where a 2 *mM* glucose concentration has been considered. Both peaks and integral area under the spectrum increase by about 36%.

**Figure 7.**
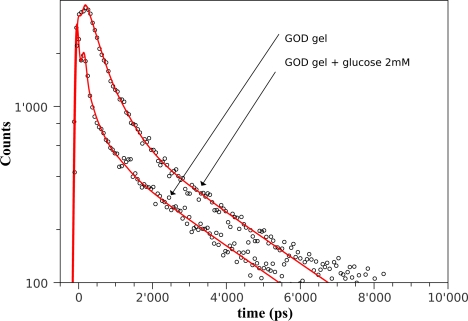
Black points are the fluorescence decays for immobilized *GOD* when excited in the FAD region (λ*_exc_* = 405 nm) without and with glucose (2 mM). Red curves are three-exponential fits. The fitting parameters are reported in Table 1.

In Figure 7 the theoretical model of Equation (1) (red curves) has been fitted with the fluorescence decay curves (black points) measured in the same experimental conditions of Figure 6.

The results of fitting are reported Table 1 showing that the immobilization matrix affects the relative contribution *f_i_* of the three fluorescent components. In particular, the first two components, *τ*_1_ and *τ*_2_, increase their fractional intensity to the detriment of the long fluorescence lifetime component *τ*_3_. It is worth noticing that the inclusion of glucose leads to the same qualitative effects observed in free *GOD*, *i.e.*, (i) an increase of the total fluorescence intensity, and (ii) a quenching of the fast and slow fluorescence components to the benefit of the intermediate one.

**Figure 8.**
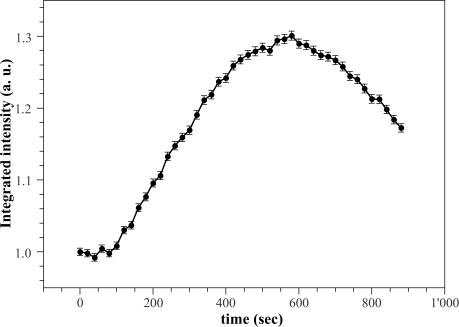
Normalized fluorescence total intensity as a function of time during the enzymatic reaction of the *GOD* entrapped in the sol gel matrix. Experimental conditions: λ*_exc_* = 405 nm, λ*_em_* according to the passband filter described in the experimental section, [*glucose*] = 2 *mM* in 0.1 *M* phosphate buffer, *pH* = 6.5.

Actually, the fluorescence enhancement lasts until the reaction cycle of Figure 2 takes place. To this regard, changes in fluorescence decay caused by the enzymatic reaction upon the addition of 2 *mM* glucose concentration, were monitored every 20 *s* with acquisition time of 20 *s*. The integral area of decay curves have been normalized to the value observed before the enzymatic reaction starts. Figure 8 shows the normalized data as a function of reaction time. The total fluorescence intensity initially remains constants and after some time it increases gradually to a maximum value of about 30% greater than that obtained in free *GOD* condition. Then, the fluorescence intensity tends to decrease gradually. Similar behaviour has been observed at any glucose concentration and has been extensively discussed in Reference [21]. This behaviour has been ascribed to the kinetics of the process, in particular to the reoxidation of *FADH*_2_. The reoxidation process proceeds at a constant rate greater than that at which the solution takes up *O*_2_ from the surrounding atmosphere (mass transfer). Initially, the reaction involves a net uptake of *O*_2_, since the mass transfer rate is very low and catalyse produces only one-half of the oxygen consumed. As the reaction proceeds, the glucose concentration and the *O*_2_ uptake decrease. The mass transfer rate becomes significant and eventually equals the oxygen uptake rate, so a steady state is reached, after which the mass transfer rate exceeds the uptake rate, and the concentration of dissolved *O*_2_ increases gradually. While the oxidation kinetics of *FADH*_2_ exceeds its production rate, the predominant species will be *FAD* and the fluorescence intensity remains constant at the beginning. As the concentration of dissolved *O*_2_ decreases, so it does the rate of *FAD* regeneration. Below a given level, *FADH*_2_ becomes the predominant species and the fluorescence intensity increases. The opposite process takes place when the concentration of *O*_2_ starts to rise again by mass transfer.

The range of the maximum fluorescence intensity is *flat* enough to allows us to measure decay curves at constant intensity; thus, in silica gel we always refer to lifetime measurements carried out at the maximum intensity.

### 3.3. Calibration Curves through Decay Curves Parameters

**Figure 9.**
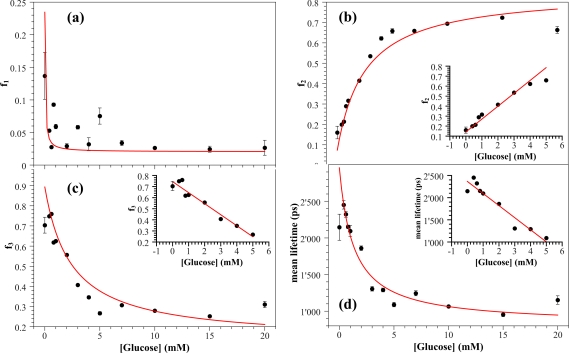
The fluorescence fractional components *f_i_* retrieved from the three-exponential fitting and the mean lifetime *τ̄* as a function of the glucose concentration. Red curves represents the fit of data with the Michaelis–Menten like expression (4). Insets in panels b,c,d show the glucose calibration curves for each parameter.

In Figure 9, fractional fluorescence intensities *f_i_* of each lifetime component and the mean lifetime *τ̄*, retrieved from fitting with decay curves, have been reported as a function of glucose concentration.

In each panel, data exhibit a Michaelis–Menten type behaviour well described by the following expression:

(4) $$y=y_{0}\pm \frac{y_{\mathrm{sat}}C}{K_{y}+C}$$

where *y* indicates the fractional component *f_i_* or the mean lifetime *τ̄*, and *C* is the glucose concentration. The quantity *y_sat_* indicates the value at saturation and *y*_0_ is the value estimated for *y* when no glucose has been added (*C* = 0) and *K_y_* is a pseudo Michaelis–Menten constant. We point out that the analysis based on the Michaelis–Menten law is usually carried out by the Equation (4) without *y*_0_, which is subtracted from the measured *y* once it is estimated at C = 0. Nevertheless, since such a procedure leads to a curve forced to cross the zero, a bias in the slope may arise. Instead, we have chosen to fit our data with Equation (4) thereby considering *y*_0_ as a fitting parameter.

The sign (−) in Equation (4) has been used for fitting *f*_1_, *f*_3_ and *τ̄* (panels a, c, and d in Figure 9), whereas the sign (+) has been used for *f*_2_ (panel b in Figure 9). The decrease of the mean lifetime *τ̄* with the glucose concentration (panel d) can be ascribed to the dynamical fluorescence quenching of the neutral form of *FAD* caused by the excited-state isomerisation from fluorescent form to non-fluorescent form [32–34].

Fitting of Equation (4) with the data reported in Figure 9 (red curves) allows us to estimate the values for *K_y_* and *y_sat_*, *i.e.*, the optokinetic parameters [31], reported in Table 2. It can be observed that the values of Michaelis–Menten constants *K_y_* estimated from decay curves are in agreement with the ones reported in the References [23,25,31]. Particularly, the estimates retrieved from the intermediate and long fluorescence components, *f*_2_ and *f*_3_, are very close to the values for free *GOD* measured by normalised reaction rates [25].

The inset in panels b–d of Figure 9 shows the range over which we have a linear relationship between the parameter *y* of the decay curves and the glucose concentration *C*:

(5) $$y=S_{y}C+y_{0}$$

The quantity *S_y_* is the sensitivity of the measurement method, and the *y*_0_ is a bias term to avoid the threshold subtraction from data. Table 2 reports the best estimates of *S_y_* from the linear fitting of Equation (5) and the range of linearity of the calibration curves. We point out that the low temporal resolution of our detection system does not allow to resolve components with lifetime less than 30∼40 ps and consequently the linear relationship between the fast fractional fluorescent component *f*_1_ and the glucose concentration cannot be investigated. Table 2 clearly shows a reduction of the linear range of about 1/4 as compared to those reported in previous work [25] where the *GOD* concentration and the thickness of the matrix were twice the values used in the present work. We wish to point out that the linear range achieved here can be easily extended as suggested in Reference [21]. The fluorescent components *f*_2_ and *f*_3_ are characterised by similar values of the sensitivity *S_y_* which gives a limit of detection (LOD) of 0.17 *mM* (applying the *S/N* = 3 criterion), whereas the LOD of the mean lifetime *τ̄* is 0.5 *mM*. Time resolved fluorescence results in a substantial improvement of the performance of the silica gel based glucose biosensor as can be seen through the comparison with the LOD value of 0.8 *mM* obtained by the steady state fluorescence measurements reported in Reference [25].

The analytical performance of various *GOD* based optical biosensors is summarised in Table 3 in terms of the immobilization support, detection method, stability, linear range and detection limits. Compared to systems making use of exogenous fluorophore and exhibiting similar linear ranges [35,36], the sensing system proposed here is characterized by higher stability and slightly lower value of LOD. On the opposite our system is expected to have a lower cost in design and synthesis since it is based on the intrinsic fluorescence of *GOD*.

## 4. Conclusions

We have presented and described a glucose sensing system that makes use of a monolithic silica gel matrix for entrapping glucose oxidase. The dynamical changes of *FAD* fluorescence occurring during the enzymatic reaction of *GOD* with glucose have been investigated by time-resolved spectroscopy. The decay curves are well described by three-exponential model (*τ*_1_, *τ*_2_ and *τ*_3_ being the fast, intermediate and long fluorescent lifetime, respectively) whose fractional intensities are strongly dependent on the glucose concentration. We have found that the overall enhancement of the fluorescence intensity observed after the glucose addition is due to the increase of the intermediate fluorescence component *τ*_2_ that results from the change of the equilibrium between open and closed conformations caused by the chemical reduction of *FAD* to *FADH*_2_. This hypothesis is confirmed by the fluorescence quenching of the fast and long components *τ*_1_ and *τ*_3_. Fluorescence quenching also indicates that the chemical reduction of *FAD* affects the photoinduced electron transfer with the surrounding aromatic residues.

Since the fractional intensities and mean lifetime are parameters strongly sensitive to the enzymatic reaction of GOD with glucose, they have been used to obtain calibration curves in view of possible application to biosensing. We have shown that the time resolved fluorescence can lead to LOD as low as 0.17 *mM* and provides a linear response from 0.4 *mM* to 5 *mM*. Compared to *GOD* based optical biosensor which makes use of exogenous fluorophore and have similar linear ranges, the proposed sensing system provides higher stability and slightly lower value of LOD. Moreover, it also has a lower cost in design and synthesis since it relies on the intrinsic fluorescence of *GOD*.

## Figures and Tables

**Table 1.** Amplitude *α_i_*, total fluorescence intensity $\sum_{i=1}^{3}\alpha_{i}$, lifetime *τ_i_*, mean lifetime *τ̄*, and relative amplitude *f_i_* of the three-exponential model for free and immobilized *GOD* in absence and in presence of glucose.

	***GOD* free**	***GOD* free + 1 mM glucose**	***GOD* immobilized**	***GOD* immobilized + 2 mM glucose**

*α*_1_	0.031 ± 0.004	0.02 ± 0.01	0.08 ± 0.01	0.037 ± 0.006
*α*_2_	0.035 ± 0.004	0.230 ± 0.003	0.094 ± 0.01	0.476 ± 0.006
*α*_3_	0.490 ± 0.003	0.790 ± 0.003	0.4 ± 0.01	0.643 ± 0.005
∑ *α*_i_	0.556	1.04	0.574	1.156
*τ*_1_ (ps)	3 ± 1	2 ± 1	8 ± 3	4 ± 2
*τ*_2_ (ps)	550 ± 60	401 ± 8	380 ± 70	450 ± 7
*τ*_3_ (ps)	3, 120 ± 40	3, 061 ± 28	2, 960 ± 190	3, 010 ± 50
*τ̄* (ps)	2,784	2,413	2,126	1,860
f_1_	0.054	0.015	0.14	0.029
f_2_	0.06	0.22	0.16	0.41
f_3_	0.88	0.76	0.70	0.56

**Table 2.** Optokinetic parameters by Michaelis-Menten fitting of the time-resolved fluorescence parameters for the immobilized *GOD*.

	**f_1_**	**f_2_**	**f_3_**	*τ̄*
**K(mM)**	0.042 ± 0.008	2.54 ± 0.06	2.40 ± 0.06	1.3 ± 0.1
**y_sat_**	0.21 ± 0.03	0.781 ± 0.002	0.764 ± 0.001	2140 ± 70 *ps*
**S_y_**		0.133 ± 0.001 *mM*^−1^	0.128 ± 0.001 *mM*^−1^	280 ± 7 *ps mM*^−1^
**Linearity (mM)**		0.4–5	0.4–5	0.4–5

**Table 3.** Analytical performance of various *GOD*-based optical biosensors.

**Immobilization support**	**Transducer**	**Stability**	**Linearity (mM)**	**LoD (mM)**	**References**
PVC solid film	CPO fluorescence	30 days	0.017–0.2	0.001	[37]
Sensing film	fluorescence	30 days	0–0.5	0.0036	[38]
Three-layer film	Qds and PtF_2_0 TPP fluorescence	?	0–3	0.2	[35]
PVA-Py matrix	pyrene groups fluorescence	30 days	0.25–3	0.19	[36]
Silica based gels	oxygen sensing film fluorescence	60 days	0.1–5	0.06	[39]
*GOD* in silica gel matrix	*f*_2_ fluorescence component of *FAD*	60 days	0.4–5	0.17	this paper
Silica gel matrix	*f*_3_ fluorescence component of *FAD*	60 days	0.4–5	0.17	this paper
Silica gel matrix	mean lifetime fluorescence of *FAD*	60 days	0.4–5	0.5	this paper
Cuprophane membrane	*FAD* fluorescence	?	2.5–10	?	[24]
Silica gel matrix	*FAD* and Tryptophan fluorescence	60 days	5–20	0.8	[25]
